# Assessment of benzodiazepine dosing in status epilepticus patients in the emergency department

**DOI:** 10.3389/fneur.2025.1645435

**Published:** 2025-10-09

**Authors:** Raniah Ibrahim Aljadeed, Amenah Alkaf, Daad Alhajlbrahim, Huda Alewairdhi, Ghadah Asaad Assiri, Rana Aljadeed, Haya Almalag, Nora Kalagi

**Affiliations:** ^1^College of Pharmacy, King Saud University, Riyadh, Saudi Arabia; ^2^Aster Sanad Hospital, Riyadh, Saudi Arabia; ^3^Hikma Pharmaceuticals KSA, Riyadh, Saudi Arabia

**Keywords:** status epilepticus, emergency department, seizure, benzodiazepines, lorazepam

## Abstract

**Background:**

Status epilepticus (SE) is a medical emergency that requires immediate care and is associated with substantial mortality and morbidity. Current guidelines recommend benzodiazepines (BZDs), regardless of the type of SE, as an initial treatment. Despite established guidelines, BZD underdosing remains common in clinical practice. This study aimed to the objective of this study was to assess BZD doses in patients administered to the ED with SE and evaluate patient outcomes in relation to BZD dosing adequacy.

**Methods:**

We conducted a single-center retrospective study of adult patients (≥18 years) who presented to the ED with SE and received BZDs from January 2021 to January 2024. Primary outcome was percent of SE patients admitted to our institution who received inadequate doses of BZDs. Secondary outcomes included ICU admission rates, need for endotracheal intubation, duration of mechanical ventilation, Glasgow Coma Scale (GCS) at discharge administration of second-line antiseizure medications, and in-hospital mortality. Demographic data, treatment details, and outcomes were collected and analyzed.

**Results:**

Among 196 adult patients included, only 17% (*n* = 34) received an adequate first dose of BZDs. Pre-hospital BZD administration occurred in 5% of cases. Lorazepam was most frequently administered (65%), followed by midazolam (20%) and diazepam (15%). Inadequate dosing rates were 77.2% for lorazepam, 90.0% for midazolam, and 96.6% for diazepam. Following first dose of BZE, the overall need for endotracheal intubation rate was 8.7% and in-hospital mortality at 3.1% across the entire cohort, with no significant differences between the 2 groups (*p* = 0.166; *p* = 0.279). The overall need for endotracheal intubation rate was 8.9%, with no statistically significant difference observed between the groups (*p* = 0.167). For patients requiring mechanical ventilation, the mean duration was 4 days (*p* = 0.988). Notably, inadequate total BZD dosing was significantly associated with increased in-hospital mortality (4.2% vs. 0%, *p* = 0.010).

**Conclusion:**

BZD underdosing in SE management remains widespread, with only 17% of patients receiving guideline-adherent initial doses. Although our study did not demonstrate significant differences in clinical outcomes based on dosing adequacy, implementation of institution-specific protocols and focused educational initiatives on weight-based BZD administration may improve guideline adherence in SE management.

## 1 Introduction

Status epilepticus (SE) is a medical emergency that requires immediate care, as the disease is associated with substantial mortality and morbidity rates (1–3). In practice, SE is defined as any seizure lasting at least 5 mins or two or more seizures in succession without a return to baseline between episodes (4, 5). Current guidelines recommend benzodiazepines as initial treatment for SE regardless of type. When administered early after seizure onset, benzodiazepines have a 79% efficacy rate in stopping seizures (6).

Benzodiazepines can be administered through various routes: midazolam is preferred for intramuscular administration, while lorazepam is preferred for intravenous (IV) administration (4, 5). The risk of refractory SE increases with prolonged seizures, making inadequate dosing of benzodiazepines potentially harmful to patient outcomes (3). Despite well-established guidelines on the appropriate benzodiazepine dosage, underdosing is quite common in clinical settings, which has led to poor seizure control and worse patient outcomes (7–11).

Previous studies have shown a widespread lack of compliance with benzodiazepine dosage. A 2017 retrospective analysis of 170 adult and pediatric emergency department (ED) patients revealed that only 11% of patients received the recommended dose of benzodiazepine; these were all pediatric patients (12). Another observational study of 44 adult patients with generalized convulsive status epilepticus (GCSE) revealed that only one patient received the adequate dose of benzodiazepine (13). Another retrospective study assessed 222 adult patients who received 403 doses of benzodiazepines. Of these, only 1.5% were treated according to recommended guidelines (9). A secondary analysis was undertaken to describe the pattern of benzodiazepine use: the Established Status Epilepticus Treatment Trial (ESETT) found that the first dose of benzodiazepine was lower than the guideline dosing recommendation for 76% of midazolam administrations and 81% of lorazepam administrations (11). A 2022 study involving 111 adult patients found that 55% of the patients did not receive an appropriate dose of benzodiazepine (10). These results highlight the propensity for underdosing benzodiazepine in patients with SE.

Several reasons may explain the widespread underdosing observed in patients with SE. Betjemann et al. (2) noted that a significant number of emergency medical service protocols in California did not adhere to evidence-based guidelines, with initial MDZ doses often being lower than those recommended. Another possible explanation is that the diagnoses had not yet been determined by the emergency department physicians in these cases. Patients may have been experiencing SE or simply recovering from recent seizures. In addition, concerns about benzodiazepine-induced respiratory depression contribute to subtherapeutic dosing levels (14–16). Alldredge et al. (17, 18) found that the rate of respiratory and circulatory adverse effects in SE patients who received a placebo was two-fold higher than in patients who received benzodiazepines. Similarly, a cross-sectional study reported that higher doses of MDZ administered by emergency medical service personnel to adult patients with SE did not lead to an increased need for respiratory support (18).

In Saudi Arabia, active epilepsy affects roughly 6.5 people per thousand (19). The substantial burden of this disease necessitates further investigation. Despite the gravity of the disease, widespread treatment errors are reported–specifically with regard to benzodiazepine dosing. To the best of our knowledge, no other studies have explored the dosing assessment of benzodiazepines for treatment of status epilepticus in Saudi Arabia. This knowledge gap makes it difficult to ascertain whether current practices align with international standards or adequately meet the needs of the Saudi population. The rationale for focusing on the Saudi context is based on the limited availability of region-specific data, which underscores the need for context-sensitive research. Moreover, variations in healthcare infrastructure, clinical practice patterns, and patient demographics may influence treatment outcomes. These factors underscore the importance of examining the local adherence to management guidelines within the Saudi healthcare setting. Therefore, the objective of this study was to assess benzodiazepine dosing in SE patients presented to the ED and assess clinical outcomes in relation to the appropriateness of benzodiazepine dosing.

## 2 Materials and methods

### 2.1 Study design and measurement

**Figure 1:**
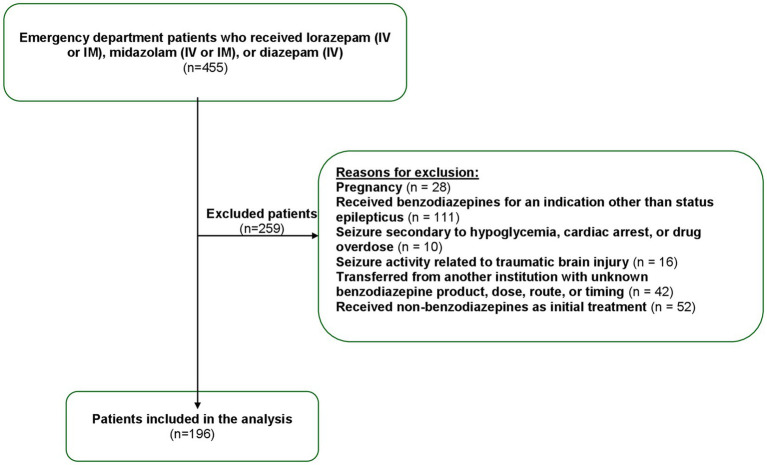
Patient flow diagram.

We conducted a single-center retrospective study assessing adult patients (≥18 years) presented to the ED with SE who received an initial dose of benzodiazepine (IV or IM lorazepam, IV or IM midazolam, or IV diazepam) between January 2021 and January 2024. We excluded patients with non-convulsive SE, as well as those who received benzodiazepine for an indication other than SE, such as those with seizures secondary to hypoglycemia, cardiac arrest, pregnancy, drug overdose, or a brain injury. Patients allergic to benzodiazepine or with incomplete medical records, as well as those transferred from another institution with an unknown benzodiazepine product, dose, route, or timing were also excluded (Figure 1).

### 2.2 Study outcomes

We extracted patient data, including demographics such as age, body mass index, sex, history of seizures, cause of seizures, and Glasgow Coma Scale scores upon ED arrival and discharge. Patient weight data was obtained from medical records, which primarily reflect weigh-ins at the time of admission. We also evaluated several clinical outcomes (intensive care unit admission, need for endotracheal intubation, duration of mechanical ventilation, in-hospital mortality secondary to seizures, and intravenous second-line antiseizure medications). The use of benzodiazepines—e.g., lorazepam, midazolam, and diazepam–for emergent treatment was documented (for both prehospital administration by the emergency medical service and in-hospital administration prior to initiating second-line antiseizure medications). Data abstraction was performed by two independent reviewers using a standardized data collection form to ensure accuracy and consistency.

The primary outcome was the number of patients with SE admitted to our institution who received inadequate benzodiazepine doses. Secondary outcomes included the relationship between adequate and inadequate doses of benzodiazepines, as well as the following: intensive care unit admission, need for endotracheal intubation, duration of mechanical ventilation, in-hospital mortality secondary to seizures, and need for intravenous second-line antiseizure medications. We divided the patients into two groups: those who received an adequate first benzodiazepine dose in the ED and those who did not. We then assessed differences in baseline demographics and secondary outcomes among these groups. A similar analysis was performed for total benzodiazepine dose adequacy.

The first and total dosages of benzodiazepine administered were converted to lorazepam equivalents (LE) based on the following ratios: 1 mg lorazepam = 2 mg midazolam = 5 mg diazepam. Based on guidelines set by the American Epilepsy Society and previous studies, inadequate dosing was defined as less than 0.1 mg/kg LE.

### 2.3 Statistical analysis

Data were coded and entered into IBM’s Statistical Package for Social Sciences (SPSS). Categorical data were presented as numbers and percentages, and continuous data were presented as means (with standard deviations) or medians (with interquartile ranges), as appropriate. Differences in categorical variables were compared using the chi-square test, and continuous variables were compared using the independent sample t-test or Mann–Whitney U test according to data distribution. The hazard ratio for primary outcomes between groups was conducted using Cox regression. Adjustment to theoretical confounders (age and gender) was done to predict the adjusted hazard ratio also using Cox regression.

## 3 Results

In total, 196 patients were included in this study. Prehospital benzodiazepine administration occurred in 5% of cases. One-hundred and twenty seven patients (64.7%) received lorazepam, 40 (20.4%) received midazolam, and 29 (14.7%) received diazepam. A total of 38.7% (*n* = 76) of the patients received at least two separate doses of benzodiazepines. The mean dose of benzodiazepine administered was 2.1 mg LE (range: 0.5–4 mg). The cumulative mean benzodiazepine dose in our cohort was 0.034 mg/kg LE (95% confidence interval, 0.031 to 0.036 mg/kg).

### 3.1 Baseline demographics of those who received adequate first dose of benzodiazepines

Among the 196 patients included in this study, 34 (17%) received an adequate first dose of benzodiazepine, while 162 (83%) did not (Table 1). Demographic analysis revealed the group to be predominantly male (60.2%) with a mean age of 32 years. The patients with history of seizure were significantly more likely to receive an adequate initial benzodiazepine dose (88.2% vs. 80.9%, *p* < 0.001). Other baseline characteristics–including the precipitating causes of seizures and Glasgow Coma Scale score at arrival–were similar between the two groups (Table 1).

**Table 1:** Baseline characteristics with stratification according to guidelines adequate first dose of Benzodiazepines (BZDs).

Characteristics		Total	Adequate 1st dose of BZD	Inadequate 1st dose of BZD	*p* value
		*N* = 196	(*N* = 34)	(*N* = 162)	
Age, mean years (SD)		32 (26)	33 (28)	28 (8)	0.700
Body mass index, mean Kilogram/meter^2^ (SD)		25.74 (6.96)	25.26 (6.32)	25.84 (7.10)	0.693
Gender (%)	Male	118 (60.2)	26 (76.5)	92 (56.8)	0.035^*^
History of seizure (%)		161 (82.1)	30 (88.2)	131 (80.9)	<0.001^*^
Precipitating cause of seizure (%)	Non-compliance	39 (19.9)	8 (24.2)	31 (19.0)	0.793
	co-exciting condition	55 (28.1)	10 (30.3)	45 (27.6)	
	Stroke	6 (3.1)	1 (3.0)	5 (3.1)	
	Malignancy	0	0	0	
	Unknown	72 (36.7)	9 (27.3)	63 (38.7)	
	Other	24 (12.2)	5 (15.2)	19 (11.7)	
Glasgow coma score at arrival, mean (SD)		11 (4)	11 (7)	10 (7)	0.085

SD, Standard deviation. ^*^Statistically significant association if *p* < 0.05.

### 3.2 Clinical outcomes of those who received adequate first dose of benzodiazepines

**Figure 2:**
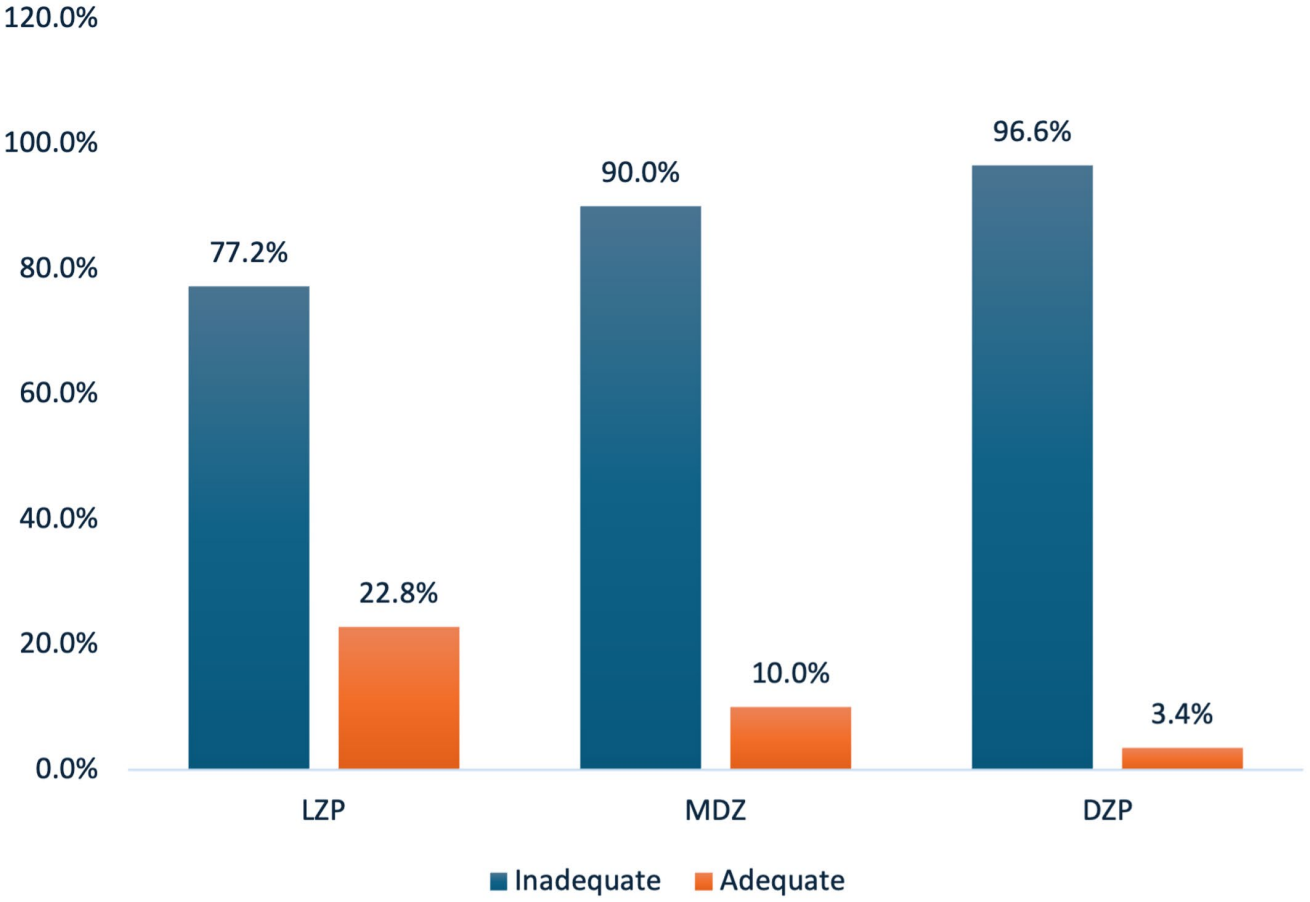
Percent of adequate first dose of benzodiazepine. LZP, Lorazepam; MDZ, Midazolam; DZP, Diazepam.

Figure 2 illustrates the percentage of adequate first doses of benzodiazepines across the three different medications. DZP showed the highest rate of inadequate dosing (96.6%), whereas MDZ showed a similar pattern, with 90% inadequate dosing. Lorazepam had the lowest inadequate dose rate (77.2%). These findings highlight a consistent gap in adherence to guideline-recommended dosing across all benzodiazepines. We divided our cohort into those who received an adequate first dose of benzodiazepines and those who did not. The secondary outcomes are listed in Table 2. The overall need for endotracheal intubation was 8.7%, with no statistically significant difference observed between the groups (*p* = 0.166). In-hospital mortality remained low (3.1%) across the entire cohort, with no significant differences between the groups (*p* = 0.279). In patients requiring mechanical ventilation, the mean duration was 4 days (*p* = 0.480). Table 2 shows the relationship between the guideline-recommended first dose of benzodiazepines and secondary outcomes. The adjusted hazard ratios for in-hospital mortality, need for endotracheal intubation, and intensive care unit admission were not statistically significant among patients who received an adequate first dose of benzodiazepines (Table 3). Lastly, the adjusted hazard ratio for in-hospital mortality with a guideline-recommended first dose of benzodiazepines was 0.586 (95% CI: 0.586–0.029, *p* = 0.728).

**Table 2:** Primary outcomes stratified by adequate first dose of BZDs with p value of differences.

Characteristics	Total	Adequate 1st dose of BZD	Inadequate 1st dose of BZD	*p* value
	*N* = 196	(*N* = 34)	(*N* = 162)	
ICU admission (%)	34 (17.6)	6 (17.6)	28 (17.6)	0.996
Need for endotracheal intubation (%)	17 (8.7)	1 (2.9)	16 (9.9)	0.166
Duration of mechanical ventilation, mean days (SD)	4 (8)	2 (2)	5 (9)	0.480
In-hospital mortality secondary to seizure (%)	6 (3.1)	2 (5.9)	4 (2.5)	0.279
Glasgow coma score at discharge, mean (SD)	14 (2)	14 (1)	14 (2)	0.257
IV second line antiepileptic drugs administered, *n* (%)	154 (78.6)	31 (20.1)	123 (79.9)	0.841
*Levetiracetam*	118 (76.6)	24 (77.4)	94 (76.4)	
*Phenytoin*	33 (21.4)	7 (22.6)	26 (21.1)	
*Valproic Acid*	1 (0.6)	0	1 (0.8)	

ICU, intensive care unit; IV, intravenous; SD, Standard deviation. ^*^Statistically significant association if *p* < 0.05.

**Table 3:** Hazard ratio of mortality, need of endotracheal intubation, and ICU admission with guidelines adequate first and total dose of BZD.

Characteristics	Hazard ratio	95% CI	*p* value	Adjusted hazard ratio^*^	95% CI	*p* value
Adequate 1st dose of benzodiazepines						
In-hospital mortality	0.515	0.046–5.742	0.589	0.586	0.586–0.029	0.728
Need of endotracheal intubation	1.453	0.312–6.756	0.643	1.581	0.333–7.506	0.564
ICU admission	0.507	0.215–1.196	0.121	0.556	0.230–1.348	0.194
Adequate total dose of benzodiazepines						
In-hospital mortality	1.174	0.191–7.227	0.863	0.137	0.005–3.737	0.293
Need of endotracheal intubation	2.207	0.759–6.415	0.146	2.942	0.917–9.441	0.070
ICU admission	0.794	0.373–1.692	0.551	0.697	0.315–1.542	0.373

ICU, intensive care unit; CI, confidence intervals; ^*^Age and gender-adjusted hazard ratio.

### 3.3 Clinical outcomes of those who received adequate total dose of benzodiazepines

Table 4 illustrates the relationship between the guideline-recommended total doses of benzodiazepines and the secondary outcomes. The overall need for endotracheal intubation was 8.9%, with no statistically significant difference observed between the groups (*p* = 0.167). In patients requiring mechanical ventilation, the mean duration was 4 days (*p* = 0.988). Notably, an inadequate total benzodiazepine dose was significantly associated with increased in-hospital mortality (4.2% vs. 0%, *p* = 0.010). The hazard ratios for in-hospital mortality, need for endotracheal intubation, and intensive care unit admission were not statistically significant among patients who received an adequate total dose of benzodiazepines (Table 3). Lastly, the hazard ratio for in-hospital mortality with a guideline-recommended total dose of benzodiazepines was 0.137 (95% CI: 0.005–3.737, *p* = 0.293).

**Table 4:** Primary outcomes stratified by adequate total dose of BZDs with *p* value of differences.

Characteristics	Total	Adequate total doses of BZDs	Inadequate total doses of BZDs	*p* value
	*N* = 192	(72)	(120)	
ICU admission (%)	34 (17.7)	16 (22.2)	18 (15.4)	0.315
Need for endotracheal intubation (%)	17 (8.9)	3 (4.2)	14 (11.7)	0.167
Duration of mechanical ventilation, mean days (SD)	4 (8)	4 (10)	4 (4)	0.988
In-hospital mortality secondary to seizure (%)	5 (2.6)	0	5 (4.2)	0.010^*^
Glasgow coma score at discharge, mean (SD)	14 (2)	14 (2)	14 (2)	0.478
IV antiepileptic drugs administered, *n* (%)	151 (78.6)	64 (88.9)	87 (75)	0.772
*Levetiracetam*	119 (61.9)	54 (75.0)	65 (56.0)	
*Phenytoin*	31 (16.1)	10 (13.9)	21 (18.1)	
*Valproic Acid*	1 (0.5)	0	1 (0.9)	

ICU, intensive care unit; IV, intravenous; SD, Standard deviation. ^*^Statistically significant association if *p* < 0.05.

## 4 Discussion

In this retrospective study of 196 patients presented to the ED with SE, only 17% received an adequate first dose of benzodiazepines. Our findings are generally consistent with results of other published trials, revealing a pattern of benzodiazepine underdosing in clinical practice (7, 9–11, 20). Sathe et al. (7) analyzed pre-enrollment data from the ESETT and found that of 102 subjects who received their first dose of benzodiazepine in the ED, only 29.8% met the minimum recommended guidelines. Similarly, Weant et al. (9) observed that only 1.5% of 222 patients who received 403 benzodiazepine doses for GCSE in the ED adhered to the guidelines. Braun et al. (10) reported that 54.7% of 111 patients with SE did not receive the recommended minimum dose of 4 mg of LE. Similarly, Kohle et al. (20) found that only 21.6% of 328 patients with SE met the current dosing guidelines. A likely reason that the underdosing rates observed in our study were higher than those reported in previous studies is the strict, weight-based parameters applied in the inadequate dosing definition. This weight-based threshold may have resulted in a more stringent classification of underdosing compared to other studies. Our study revealed higher rates of inadequate dosing: 96.6, 90, and 77.2% for diazepam, midazolam, and lorazepam, respectively. Similarly, Sathe et al. (11) examined 460 initial benzodiazepines doses administered to patients with SE and found that 76% of midazolam and 81% of lorazepam administrations were below the recommended doses. This pattern of systematic underdosing persists across multiple geographical regions. Possible explanations for the widespread benzodiazepine underdosing in SE may stem from multiple factors, including delayed recognition of SE, uncertainty regarding the applicability of dosing recommendation guidelines across SE subtypes, and limited familiarity with weight-based dosing in adults. Furthermore, clinicians are concerned about adverse effects, such as respiratory depression and oversedation. Of all treatments evaluated, lorazepam showed the lowest rate of inadequate dosing (77.2%). This may be explained by its frequent use and greater familiarity among providers. In our study, lorazepam was also the most commonly administered benzodiazepine (64.7% of cases), likely contributing to better adherence to recommended dosing guidelines.

The mean duration of mechanical ventilation after the first dose of benzodiazepine was 4 days with no significant differences between the groups. However, this contrasts the findings of Kohle et al. (20), which revealed that patients with GCSE benefited from adequate benzodiazepines dosing due to significantly shortened mechanical ventilation durations (37.1 vs. 208 h). This discrepancy may be explained by differences in the demographics of the study population. Our cohort had a relatively young mean age (32 years) and a high prevalence of a seizure history (82.1%). Factors such as age and SE severity are strong predictors of functional SE outcomes, potentially obscuring the risks that might emerge with benzodiazepine underdosing (20).

Notably, results showed that inadequate total benzodiazepine dosage was significantly associated with increased in-hospital mortality rates (*p* = 0.01). Variations in patient populations and dosing standard interpretations could explain such discrepancies compared from previous studies (10, 11, 20). However, these findings should be interpreted with a degree of caution as further prospective studies are necessary to draw substantive conclusions. In addition, there is no strong evidence to support a statistically significant association between guideline-appropriate benzodiazepine dosing, whether first dose or total dose, and in-hospital mortality. Further prospective studies with larger sample sizes are needed to confirm these findings and provide more definitive conclusions.”

The consistent finding of widespread benzodiazepine underdosing in SE management in our cohort and across multiple studies underscores the urgent need for interventions to enhance adherence to the established dosing guidelines. This is particularly critical, as it suggests a link between inadequate benzodiazepine dosing and poor clinical outcomes. These findings highlight several high-priority targets for quality improvement initiatives, which may include development of institution-specific treatment protocols and rigorous implementation of standardized, evidence-based dosing protocols that clearly define appropriate dosing. In addition, healthcare institutions must establish clear policies mandating adherence to these protocols, fostering interdisciplinary collaboration and integrating clinical decision support within electronic health records to prevent under-dosing and medication errors. Lastly, ongoing provider education on appropriate dosing of benzodiazepines can help ensure competency and cultivate a culture of medication safety.

This study had several limitations. First, the fact that it was a single-center, retrospective design limits our ability to generalize from the findings. The study was conducted at a tertiary academic medical center in Saudi Arabia. However, further studies involving larger, more diverse populations from multiple centers across Saudi Arabia are warranted to corroborate these results. In addition, as it relies on the ED physician’s diagnosis at the time of therapy, some patients may have experienced non-epileptic events. However, this does not affect the primary objective of assessing adherence to the benzodiazepine guidelines. Furthermore, because of the retrospective nature of the study, the dosing and sequence of medication administration may have been skewed during the post-event documentation process. The potential inaccuracy of patient weight data (as some weights may have been estimated rather than directly measured), may have influenced subsequent dosage classifications. Lastly, the etiology of status epilepticus remains an important prognostic factor that may influence treatment outcomes; as such, it warrants further research.

In conclusion, our findings suggest that benzodiazepine underdosing in SE remains a common phenomenon in patients with SE. Appropriate benzodiazepine dosing may be achieved through the implementation of institution-specific protocols and focused educational initiatives on weight-based administration of benzodiazepines.

## Data Availability

The datasets presented in this article are not readily available because access to the dataset is restricted due to institutional policies. Requests to access the datasets should be directed to RIA, raaljadeed@ksu.edu.sa.
